# Quantifying dose uncertainties resulting from cardiorespiratory motion in intensity-modulated proton therapy for cardiac stereotactic body radiotherapy

**DOI:** 10.3389/fonc.2024.1399589

**Published:** 2024-07-08

**Authors:** Weige Wei, Zhibin Li, Qing Xiao, Guangyu Wang, Haiping He, Dashuang Luo, Li Chen, Jing Li, Xiangyu Zhang, Taolin Qin, Ying Song, Guangjun Li, Sen Bai

**Affiliations:** ^1^ Department of Radiation Oncology, Cancer Center, West China Hospital, Sichuan University, Chengdu, Sichuan, China; ^2^ Department of Radiotherapy Physics & Technology, West China Hospital, Sichuan University, Chengdu, Sichuan, China; ^3^ Department of Radiotherapy & Oncology, The First Affiliated Hospital of Soochow University, Institute of Radiotherapy & Oncology, Soochow University, Suzhou, China; ^4^ State Key Laboratory of Oncology in South China, Guangdong Provincial Clinical Research Center for Cancer, Sun Yat-sen University Cancer Center, Guangzhou, China; ^5^ Department of Radiotherapy & Oncology, The Second Affiliated Hospital of Soochow University, Suzhou, China; ^6^ Department of Medical Physics, Brown University, Providence, RI, United States

**Keywords:** cardiac stereotactic body radiotherapy, IMPT, 4D dose reconstruction, interplay effects, cardiorespiratory motion

## Abstract

**Background:**

Cardiac stereotactic body radiotherapy (CSBRT) with photons efficaciously and safely treats cardiovascular arrhythmias. Proton therapy, with its unique physical and radiobiological properties, can offer advantages over traditional photon-based therapies in certain clinical scenarios, particularly pediatric tumors and those in anatomically challenging areas. However, dose uncertainties induced by cardiorespiratory motion are unknown.

**Objective:**

This study investigated the effect of cardiorespiratory motion on intensity-modulated proton therapy (IMPT) and the effectiveness of motion-encompassing methods.

**Methods:**

We retrospectively included 12 patients with refractory arrhythmia who underwent CSBRT with four-dimensional computed tomography (4DCT) and 4D cardiac CT (4DcCT). Proton plans were simulated using an IBA accelerator based on the 4D average CT. The prescription was 25 Gy in a single fraction, with all plans normalized to ensure that 95% of the target volume received the prescribed dose. 4D dose reconstruction was performed to generate 4D accumulated and dynamic doses. Furthermore, dose uncertainties due to the interplay effect of the substrate target and organs at risk (OARs) were assessed. The differences between internal organs at risk volume (IRV) and OAR_real_ (manually contoured on average CT) were compared. In 4D dynamic dose, meeting prescription requirements entails V_25_ and D_95_ reaching 95% and 25 Gy, respectively.

**Results:**

The 4D dynamic dose significantly differed from the 3D static dose. The mean V_25_ and D_95_ were 89.23% and 24.69 Gy, respectively, in 4DCT and 94.35% and 24.99 Gy, respectively, in 4DcCT. Eleven patients in 4DCT and six in 4DcCT failed to meet the prescription requirements. Critical organs showed varying dose increases. All metrics, except for D_mean_ and D_50_, significantly changed in 4DCT; in 4DcCT, only D_50_ remained unchanged with regards to the target dose uncertainties induced by the interplay effect. The interplay effect was only significant for the D_max_ values of several OARs. Generally, respiratory motion caused a more pronounced interplay effect than cardiac pulsation. Neither IRV nor OAR_real_ effectively evaluated the dose discrepancies of the OARs.

**Conclusions:**

Complex cardiorespiratory motion can introduce dose uncertainties during IMPT. Motion-encompassing techniques may mitigate but cannot entirely compensate for the dose discrepancies. Individualized 4D dose assessments are recommended to verify the effectiveness and safety of CSBRT.

## 1 Introduction

Cardiovascular arrhythmia is one of the most common cardiovascular diseases, with tachyarrhythmia being the leading cause of sudden cardiac mortality. In 2020, 17.9 million people died of cardiovascular diseases, comprising 31% of global deaths. Various treatments are available for tachyarrhythmia; however, they have poor effectiveness and severe complications (1, 2).

Cardiac stereotactic body radiotherapy (CSBRT) is a treatment option for refractory arrhythmias (3). Stereotactic body radiotherapy (SBRT) targets well-demarcated, limited-volume malignant or benign tumors using image guidance and single or fractionated high-dose irradiation (4). CSBRT is primarily implemented using CyberKnife or C-arm linear accelerators with high-energy X-rays. Nearly all CSBRT cases involved administering a single 25-Gy fraction. A possible mechanism of CSBRT is inducing alterations in the electrical activity of the arrhythmia substrate and promoting partial myocardial fibrosis (5, 6). The efficacy and safety of early CSBRT for refractory arrhythmia have been reported (7, 8).

Owing to its distinct characteristics, proton therapy improves dose distribution for tumors and adjacent normal tissues, providing benefits in pediatric cases and tumors in complex anatomical locations compared with conventional therapies (9, 10). Therefore, its use for cardiovascular diseases is promising. The Bragg peak at the end of the proton range allows selective deposition of the maximum dose within the target volume. In contrast, photons can achieve similar consistency only by cross-firing many fields. Photons expose large volumes of normal tissue to low or medium-dose levels. In intensity-modulated proton therapy (IMPT), beams can be safely bent around complex critical structures, sparing them without compromising target coverage (11).

However, dose uncertainties due to respiration remain a concern in SBRT for thoracic and abdominal lesions. Hypofractionated irradiation is more susceptible to respiration than conventional fractionation (12, 13). The interplay effects of dynamic delivery and tumor motion degrade the plan quality, particularly in IMPT (14). The degree of motion-induced dose uncertainty depends on the delivery system and varies according to spot size (15), fractionation (13), delivery time (16), scanning technique (17), and patient motion amplitude (18).

Dose uncertainties resulting from cardiorespiratory motion are inevitable during CSBRT. Inadequate irradiation doses to the substrate target and overdoses to normal tissues may lead to failed cardiac ablation and adverse effects. Notably, cardiorespiratory motion management techniques for CSBRT have not been established, and the relevance of conventional motion-encompassing techniques and margins for target volume remains unclear. Furthermore, four-dimensional computed tomography (4DCT) has limitations in visualizing cardiac substructure and motion characteristics. Retrospective electrocardiographically gated 4D cardiac CT (4DcCT) should be used to assess cardiac motion characteristics and dose uncertainties caused by cardiac pulsation (19).

Therefore, this study evaluated the interplay effects of cardiorespiratory motion on IMPT dosimetric parameters and determined whether cardiorespiratory motion-encompassing methods in IMPT can compensate for dose discrepancies caused by cardiorespiratory motion.

## 2 Methods

### 2.1 Patient selection

We retrospectively included 12 patients with refractory arrhythmias who underwent CSBRT via photon therapy between April 2021 and December 2022. 4DcCT and 4DCT (GE, Revolution ES, USA) images were acquired before CSBRT and divided into 10 phases (0%–90% of the breathing circle and R-R electrocardiography [ECG] interval) (19). The concept of clinical target volume (CTV) was used to define substrate targets. The CTV and organs at risk (OARs) were delineated and confirmed by cardiologists and radiation oncologists in the 4DcCT 0% phase using enhanced scans. The ethics committee of West China Hospital approved the use of patient data.

### 2.2 Treatment planning

All proton plans were simulated in our treatment planning system, RayStation (version 12A, Raysearch, Stockholm, Sweden). The treatment delivery machine used was the cyclotron-based accelerator system, IBA ProteusPLUS. The beam spot size sigma varies between 2.6 and 7.2 mm depending on the proton energy (230 to 70 MeV). The minimum and maximum spot weights were 0.02 and 4 MU, respectively. The dose grid had a 2-mm resolution in all directions; the dose engine was a Monte Carlo V5.4 with a 0.5% uncertainty for the proton plans.

#### 2.2.1 Target and OAR definition

**Figure 1:**
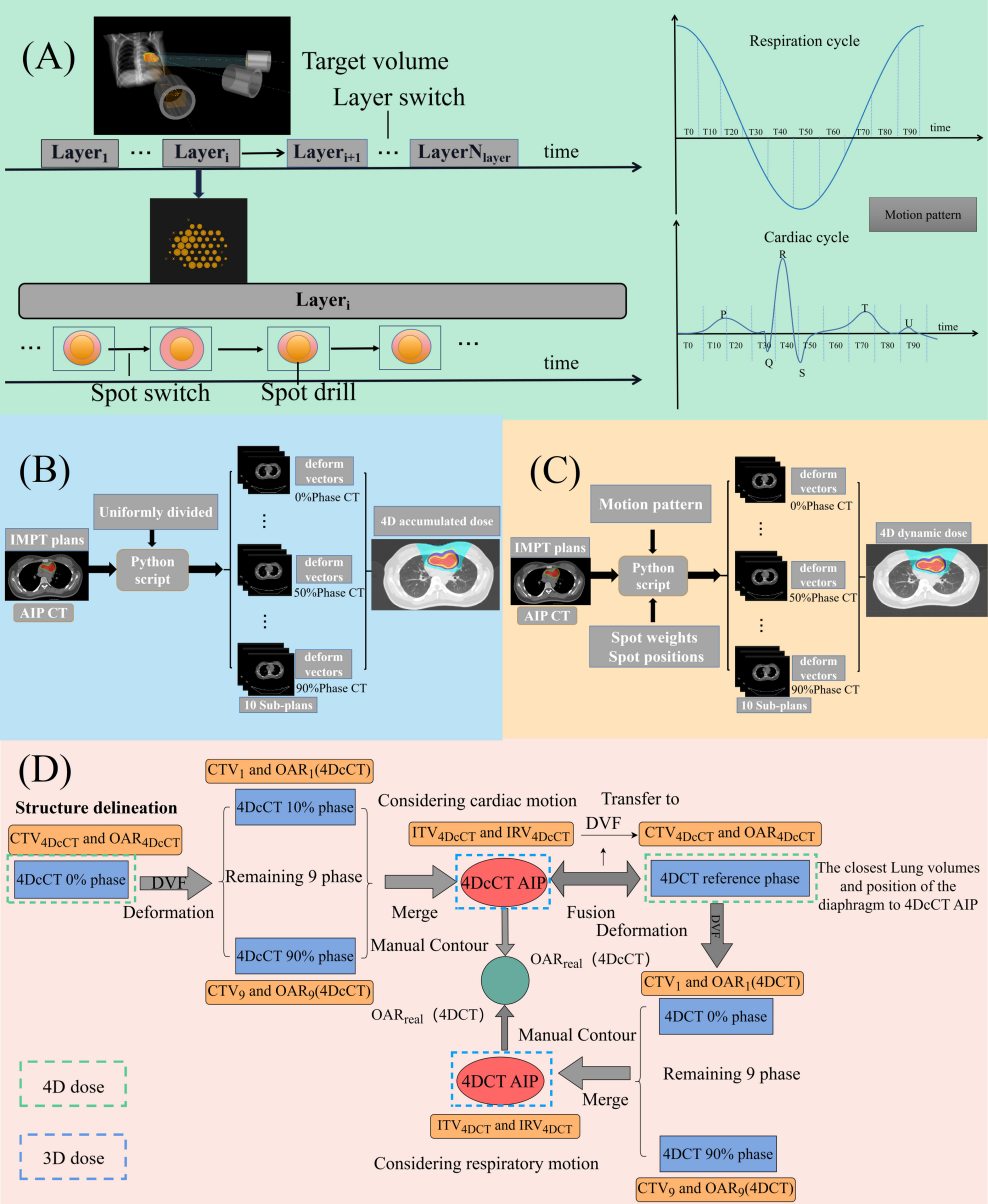
**(A)** Time structure of proton treatment delivery and cardiorespiratory motion cycle; **(B)** process of 4D accumulated dose reconstruction; **(C)** process of 4D dynamic dose reconstruction; and **(D)** process of target and OAR definition in CSBRT.

Cardiologists and radiation oncologists collaborated to delineate the CTV in the 0% phase of the 4DcCT. OARs include cardiac substructures such as the left atrium (LA), right atrium (RA), aorta (AO), left ventricle (LV), right ventricle (RV), left ventricular wall (LVW), pulmonary arteries (PA), esophagus (ESO), spinal cord, lungs, and heart excluding the CTV (HE). The internal target volume (ITV) and internal organ-at-risk volume (IRV) were generated using motion-encompassing techniques as follows (20, 21): After delineating CTV_4DcCT_ and OAR_4DcCT_ in the 4DcCT 0% phase was completed, the structures were deformed and propagated to the remaining nine phases using MIM 5.2 (MIM Software Inc, USA); subsequently, the average intensity projection (AIP) CT of 4DcCT was generated. Radiation oncologists and cardiologists verified the deformed structures on 4DcCT. The structures of the 10 phases were mapped to the AIP CT and merged to generate ITV_4DcCT_ and IRV_4DcCT_, considering the cardiac motion. Cardiologists and radiation oncologists redelineated OARs on the AIP image and standardized the names as OAR_real_ to distinguish them from the IRVs. For 4DCT, the 4DcCT AIP image of each patient was fused and compared with the 10 phases of 4DCT. The 4DCT phase with the closest lung volume and diaphragmatic position to the AIP CT of 4DcCT was selected as the reference phase. ITV_4DcCT_ and IRV_4DcCT_ were transferred to the 4DCT reference phase and renamed CTV_4DCT_ and OAR_4DCT_. The structures of the 4DCT reference phase were deformed into the remaining nine phases; the same operation was repeated to yield ITV_4DCT_ and IRV_4DCT_. Subsequently, a total of 240 time-phase and 24 AIP CT images from 12 patients, along with their corresponding structures, were imported into the Raystation planning system for further planning. All plans were designed based on ITV instead of PTV while ignoring setup errors to visualize cardiorespiratory motion effects comprehensively (**Figure 1D**).

#### 2.2.2 Plan design and optimization

All the simulated 3D static plans were designed based on the AIP images of 4DcCT and 4DCT. The proton plans comprised three or four coplanar fields delivered using IBA pencil beam scanning. Beam angles were carefully selected to reduce the radiation exposure of normal tissues according to the target location, ensuring that the distal edge was strategically positioned away from critical structures. The relative biological effectiveness (RBE) parameter for proton plans was set to 1.1, and a 25-Gy (RBE) prescription dose was delivered to 95% of the ITV in a single fraction. This study primarily aimed to investigate the effect of cardiorespiratory motion on the dose metrics for substrate targets. However, sacrificing the dose to OARs when they were close to or within the ITV is conventional. Furthermore, the dose to the OARs should be minimized while meeting the dose requirements of the ITV. The rescanning technique and other robust optimization methods were avoided to fully visualize the cardiorespiratory motion effects. All plans were normalized to a 100% prescribed dose, covering 95% of the ITV.

### 2.3 4D dose reconstruction

4D doses can be classified into two types. The first is the 4D-accumulated dose, which accounts for spots uniformly distributed across 10 phases during treatment and is largely affected by the dose-blurring effect. The second type is the 4D dynamic dose, which is based on the temporal structure of the delivery, with spots assigned sequentially to the corresponding phase, and is primarily influenced by dose blurring and interplay effects (22–25). The deformation vector fields (DVFs) between the reference phase and the remaining phases of the 4DCT and 4DcCT were generated using a deformable registration algorithm. The deformable image registration (DIR) algorithm used for dose warping, Anatomically Constrained Deformation Algorithm (ANACONDA), adopts a hybrid approach combining intensity and geometric-based algorithms (26). This algorithm has been rigorously validated and effectively implemented in various thoracic (27, 28) and cardiac applications (29), demonstrating its effectiveness in handling complex motions. Furthermore, Sarudis et al.’s (30)investigation into the accuracy of deformable image registration for thoracic CT images indicated that the average Dice coefficient for cardiac registration surpassed 0.9, consistent with the TG 132 guidelines (31). Additionally, **Supplementary Figure S4** provides a detailed view of the deformation registration employed in our research.

To calculate the 4D accumulated dose, 3D static plans were transplanted into all phases of 4DCT and 4DcCT. Using the DVFs from the 4DCT reference phase or 4DcCT 0% phase to all other phases, the dose distributions from all phases were transferred to and averaged over the reference or 0% phase to create a composite dose for the entire respiratory or cardiac cycle (**Figure 1B**).

For the 4D dynamic dose, a beam-time model was built considering the beam delivery parameters, such as the energy layer switching, spot switching, and spot drilling times. Our study adopted the time–structure modeling method of the IBA cyclotron proposed by Zhao et al., which could accurately predict the delivery sequence of the accelerator (32). A constant breathing cycle of 4 s and a cardiac cycle of 0.8 s (heart rate: 75 bpm) were assumed without considering asymmetric motion patterns, while all plans were set to commence at the 0% phase of the motion cycle. The 3D static plans were split into 10 sub-plans using in-house Python scripts that retrieved information from the time structure (**Figure 1A**). Each subplan contained only spots corresponding to the associated phase of the motion cycle. The 4D dynamic dose accumulation in each available 4DCT or 4DcCT was performed by warping the subplan dose distributions per phase onto the reference or 0% phase via DVFs from ANACONDA. The phase-specific warped doses were subsequently summed on the reference or 0% phase CT images to generate the final dose (**Figure 1C**).

### 2.4 Plan quality evaluation

Our study employed a 4D dose evaluation method and compared the metrics for 3D static (ITV/IRVs) and 4D dynamic (CTV/OARs) doses (33). Discrepancies between the 4D accumulated and dynamic doses were analyzed to explore interplay effects. Dosimetric differences between IRV and OAR_real_ were determined to assess the accuracy of the OAR dose evaluation. The plan quality metrics used are as follows:

Conformity Index (CI), defined by Equation (1):


(1) $$\begin{array}{c} CI=\frac{V_{ITV_{\_25Gy}}^{2}}{V_{ITV}\times V_{25Gy}} \end{array}$$

V_ITV_25Gy_ refers to the intersection between the ITV and the region receiving ≥25 Gy.

Homogeneity Index (HI), defined by Equation (2):


(2) $$\begin{array}{c} HI=\frac{D_{2}-D_{98}}{D_{mean}} \end{array}$$

Gradient Indexes (GIs), defined by Equations (3–5):


(3) $$\begin{array}{c} GI=\frac{V_{12.5\text{Gy}}}{V_{25\text{Gy}}} \end{array}$$

(4) $$\begin{array}{c} GI_{high}=\frac{V_{18.75\text{Gy}}}{V_{25\text{Gy}}} \end{array}$$

(5) $$\begin{array}{c} GI_{low}=\frac{V_{6.25\text{Gy}}}{V_{25\text{Gy}}} \end{array}$$

Dose-volume histograms (DVHs) were generated for all doses. We calculated the DVH-based dosimetric indices for the substrate target and the OARs, including the heart, cardiac substructures, lungs, esophagus, and spinal cord. The dosimetric parameters considered were the dose covering a certain volume percentage of the structure (D_%_) and the percentage volume of the structure receiving doses >x Gy (V_x_). The V_x_ was expressed as absolute volumes (cc) in the OARs. The 3D static dose collected the dose metrics of the ITV and IRV and the manually contoured OAR_real_ on the AIP images. The 4D doses were used to count the dosimetry metrics of the CTV and OARs on the reference or 0%-phase CTs.

### 2.5 Statistical analysis

The data were analyzed using SPSS software (version 26; IBM, Armonk, NY, USA). The Wilcoxon signed-rank test was used to compare differences in the dose metrics. Statistical significance was set at *p<* 0.05, with a more stringent threshold of *p*< 0.01 denoting a higher level of statistical significance.

## 3 Results

### 3.1 Cardiorespiratory motion-induced dose uncertainty in substrate target

#### 3.1.1 Comparison of 4D dynamic dose and 3D static dose in the target

The 4D dynamic dose considers dose blurring and interplay effects during cardiorespiratory motion, reflecting the actual dose received during treatment. Respiratory motion introduces significant dose discrepancies during treatment compared with cardiac pulsation. In our institution, it is essential to achieve V_25_ and D_95_ values of 95% and 25 Gy, respectively, in the 4D dynamic dose. This is because all plans are normalized to ensure that 95% of the target volume receives the prescribed dose. According to **Table 1**, in 4DCT, 11 of the 12 patients failed to meet the acceptability criteria, with the lowest V_25_ and D_95_ being only 79.3% and 24.13 Gy, respectively. In 4DcCT, 6 of 12 patients could not fulfill the prescription requirements, with the lowest V_25_ and D_95_ being only 89.5% and 24.76 Gy.

**Table 1:** Metrics between 3D dose and 4D dynamic dose in substrate target.

Metrics	4DCT			4DcCT		
	3D dose	4D dynamic dose	Discrepancy	3D dose	4D dynamic dose	Discrepancy
V_25_ (%)	95.00±0.00	89.23±1.50	5.77±5.18**	95.00±0.00	94.35±1.35	0.65±3.11
D_99_ (Gy)	24.18±0.39	23.87±0.63	0.31±0.80	24.53±0.28	24.57±0.25	-0.04±0.37
D_98_ (Gy)	24.59±0.20	24.28±0.40	0.32±0.49	24.77±1.44	24.78±0.19	-0.01±0.25
D_95_ (Gy)	25.00±0.00	24.69±0.28	0.31±0.28**	25.00±0.00	24.99±0.14	0.01±0.14
D_50_ (Gy)	25.72±0.21	25.70±0.22	0.02±0.14	25.58±0.17	25.63±0.20	-0.05±0.11*
D_2_ (Gy)	26.72±0.30	26.93±0.40	-0.21±0.36**	26.59±0.23	26.62±0.26	-0.03±0.19
D_1_ (Gy)	26.83±0.29	27.11±0.44	-0.28±0.41*	26.70±0.23	26.73±0.26	-0.03±0.19
D_max_ (Gy)	27.24±0.29	27.78±0.98	-0.54±1.03*	27.05±0.16	27.10±0.33	-0.04±0.25
D_min_ (Gy)	19.35±2.60	19.86±2.18	-0.51±2.31	20.68±1.65	21.04±1.65	-0.35±1.48
D_mean_ (Gy)	25.73±0.19	25.69±0.21	0.04±0.13	25.62±0.15	25.65±0.15	-0.03±1.21
CI	0.88±0.04	0.63±0.11	0.25±0.11**	0.87±0.43	0.66±0.10	0.21±0.10**
HI	0.08±0.02	0.10±0.03	-0.02±0.03*	0.07±0.13	0.07±0.01	-0.01±0.01
GI	3.41±0.54	3.95±0.48	-0.54±0.29**	3.84±0.42	4.27±0.56	-0.43±0.22**
GI_high_	2.01±0.24	2.30±0.24	-0.29±0.15**	2.22±0.20	2.43±0.27	-0.22±0.12**
GI_low_	6.64±1.80	7.73±2.02	-1.09±0.68*	7.79±1.75	8.70±2.00	-0.91±0.50**

4DCT, Four-dimensional computed tomography; 4DcCT, Four-dimensional cardiac computed tomography; CI, Conformity index; GI, Gradient index; HI, Homogeneity index.

For the target metrics, values are presented as mean±standard deviation. Discrepancy: Difference between the 3D and 4D dynamic doses (discrepancy=3D dose−4D dynamic dose). A single asterisk (*) indicates a p-value less than 0.05, signifying statistical significance, whereas a double asterisk (**) denotes a p-value less than 0.01, indicating a higher level of statistical significance.

Motion caused an increase in hot spots in the substrate target, with a statistical difference observed only in respiratory motion. In 4DCT, D_max_ increased by 0.67 ± 0.95 Gy (*p<* 0.05), leading to poorer homogeneity in the substrate target and a decreased dose -fall-off gradient. Additionally, in 4DCT, the HI and GI of the 4D dynamic dose increased, with changes as follows: HI increased by 0.02 ± 0.03 (*p<* 0.05), and the GI by 0.54 ± 0.29 (*p<* 0.01). In 4DcCT, owing to less pronounced cardiac pulsation effects, only the GI showed increases by 0.43 ± 0.22 (*p<* 0.01).

Overall, ITV-based motion-encompassing techniques were ineffective in mitigating the dose discrepancies caused by cardiorespiratory motion.

#### 3.1.2 Evaluation of dose uncertainties induced by interplay effects in the target

**Figure 2:**
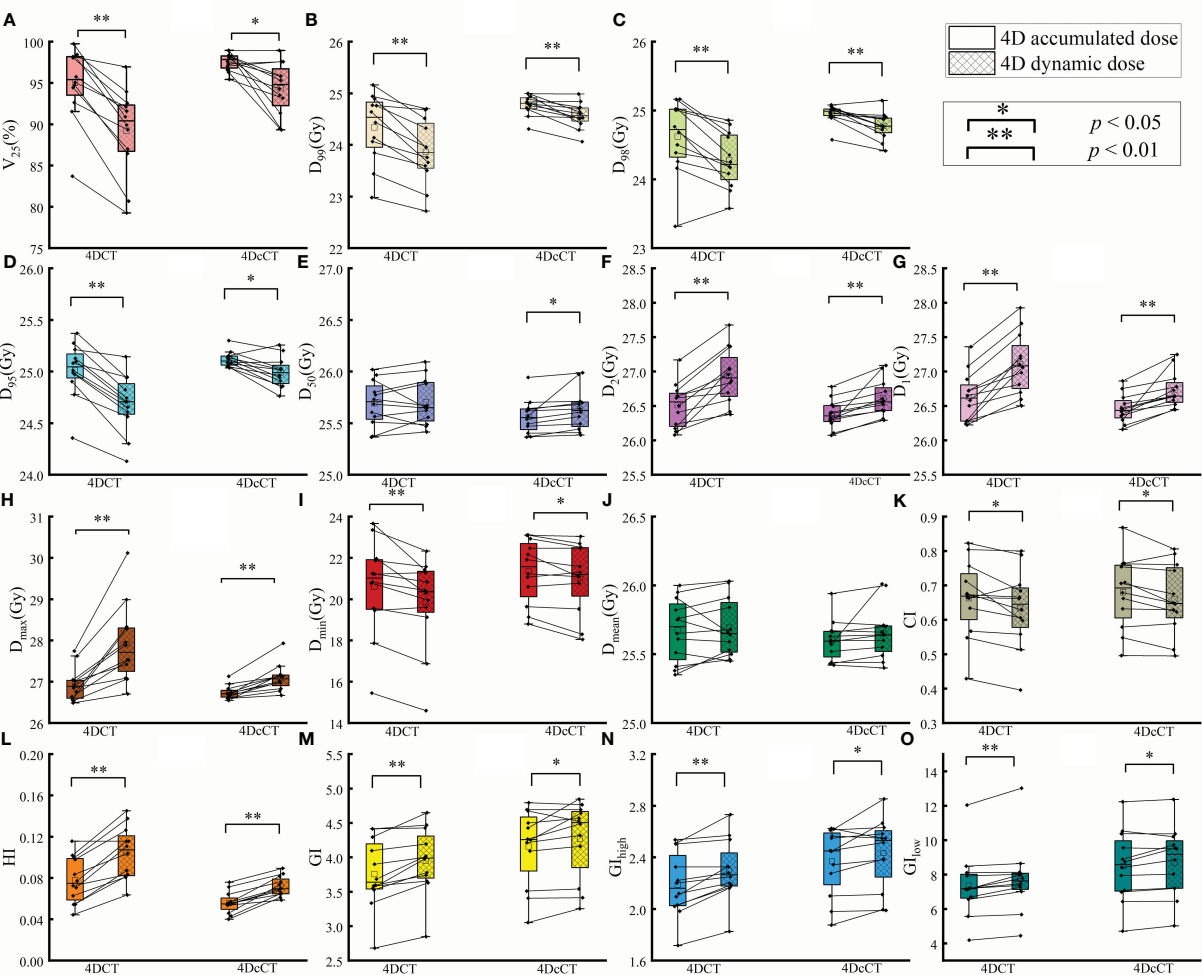
Comparison of dose metrics between 4D accumulated and 4D dynamic doses for substrate target. The figure presents the differences in dose metrics, including **(A)** V_25_, **(B)** D_99_, **(C)** D_98_, **(D)** D_95_, **(E)** D_50_, **(F)** D_2_, **(G)** D_1_, **(H)** maximum dose (D_max_), **(I)** minimum dose (D_min_), **(J)** mean dose (D_mean_), **(K)** Conformity Index (CI), **(L)** Homogeneity Index (HI), **(M)** Gradient Index (GI), **(N)** High Dose Gradient Index (GI_high_), and **(O)** Low Dose Gradient Index (GI_low_). A single asterisk (*) indicates a *p*-value less than 0.05, signifying statistical significance, whereas a double asterisk (**) denotes a *p*-value less than 0.01, indicating a higher level of statistical significance.

**Figure 3:**
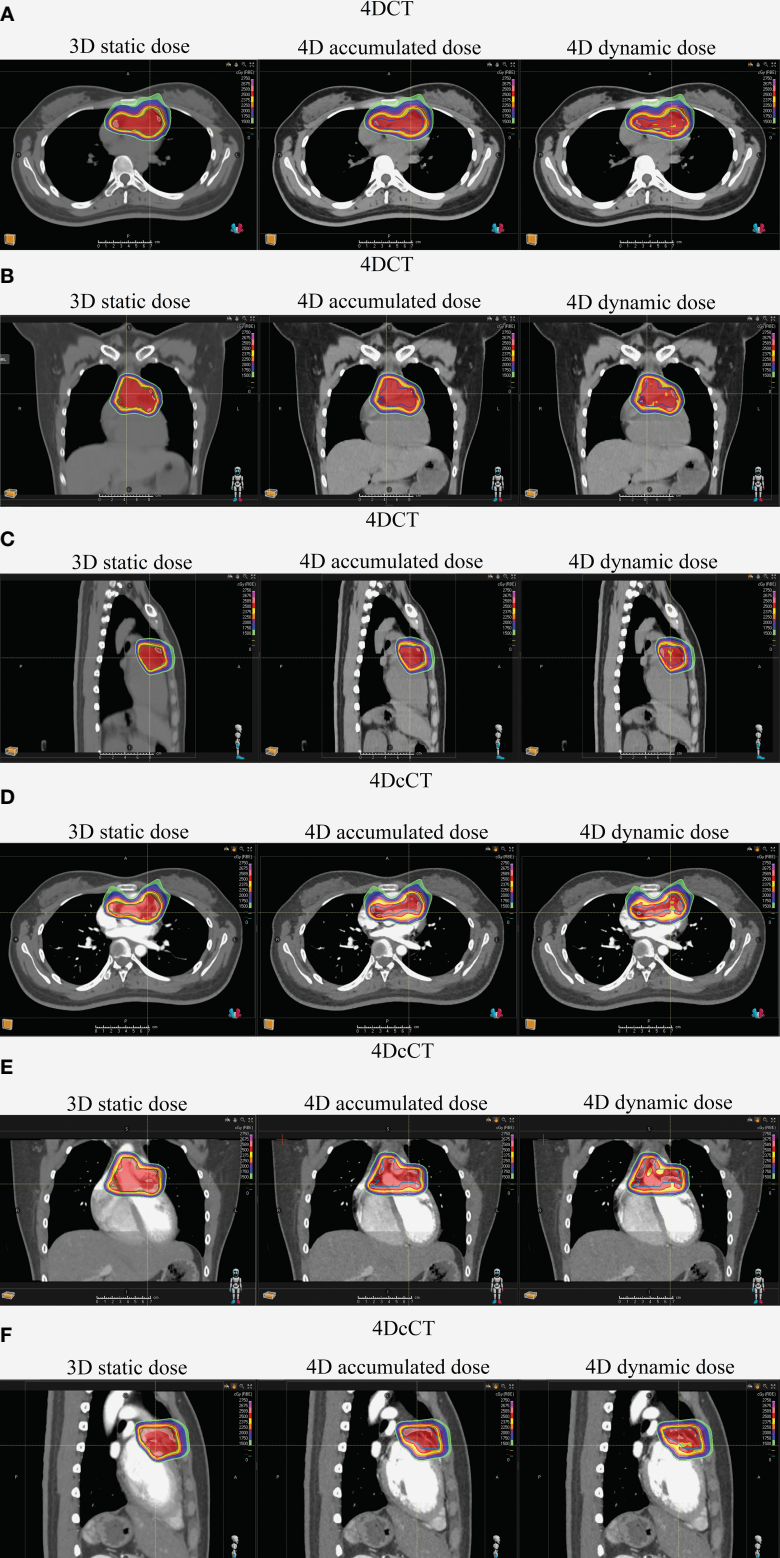
Dose distribution comparison for one patient across 3D static and 4D accumulated and dynamic doses using 4DCT and 4DcCT. Panels illustrate various anatomical views: **(A)** transverse in 4DCT, **(B)** coronal in 4DCT, **(C)** sagittal in 4DCT, **(D)** transverse in 4DcCT, **(E)** coronal in 4DcCT, and **(F)** sagittal in 4DcCT.

The comparison between 4D accumulated and dynamic doses highlighted the interplay effects caused by cardiorespiratory motion, with significant variations observed in most metrics (**Figure 2**). Generally, cardiorespiratory motion leads to increased high doses and decreased low doses within the target, indicating the formation of cold spots and hot spots. **Figure 3** also well demonstrates the presence of cold and hot spots in the target. In 4DCT, average decreases included 5.81% for V_25_ and 0.47 Gy, 0.34 Gy, 0.30 Gy, and 0.74 Gy for D_99_, D_98_, D_95_, and D_min_, respectively (*p<* 0.01). Conversely, D_max_ experienced average increases of 0.97 Gy (*p*< 0.01). Interplay effects significantly deteriorated the plan quality. The CI decreased by 0.03 ± 0.04 (*p<* 0.05), whereas HI and GI increased by 0.03 ± 0.01 (*p<* 0.05), 0.19 ± 0.14 (*p<* 0.01), respectively.

Similarly, dose uncertainties in 4DcCT owing to cardiac pulsation were significant, yet they were less substantial compared with those induced by respiratory motion. The average reductions observed were 3.22% for V_25_ (*p*< 0.05), 0.21 Gy for D_99_ (*p*< 0.01), 0.18 Gy for D_98_ (*p*< 0.01), 0.13 Gy for D_95_ (*p*< 0.05), and 0.31 Gy for D_min_ (*p*< 0.05). Conversely, increases were noted in D_max_ by 0.36 Gy (*p*< 0.01). The CI decreased slightly by 0.02 ± 0.03 (*p<* 0.05). HI and GI increased by 0.02 ± 0.01 (*p<* 0.01), 0.11 ± 0.17 (*p<* 0.05), respectively.

### 3.2 Cardiorespiratory motion-induced dose uncertainty in the OARs

#### 3.2.1 Comparison of 4D dynamic dose and 3D static dose in the OARs

Currently, the dose constraints for the cardiac substructures and critical organs in CSBRT are not well-defined. However, if the OAR metrics in the 4D dynamic dose exceeded those in the 3D static dose, the IRVs could not evaluate the actual dose of the OARs, leading to overdoses, and the corresponding plans failed to meet the acceptability criteria.

As shown in **Table 2**, in 4DCT, the D_50_ of LV, LVW, LA, RA, RV, and HE in the 4D dynamic dose was significantly higher than that in the 3D static dose (*p<* 0.05). D_50_ also increased in 4DcCT; however, the magnitude was relatively small, and only the HE had a significant increase (*p<* 0.05). IRVs tend to underestimate the D_50_ in 4D dynamic doses owing to their consideration of the larger volumes involved in cardiorespiratory motion, typically resulting in lower D_50_ values. This underestimation may reveal limitations in using IRVs to evaluate volume-related metrics.

**Table 2:** Metrics between 3D dose and 4D dynamic dose in the OARs.

Metrics		4DCT			4DcCT		
		3D dose	4D dynamic dose	Discrepancy	3D dose	4D dynamic dose	Discrepancy
LA	D_50_ (Gy)	0.09(0.00-1.87)	0.16(0.00-4.30)	-0.29±0.72*	0.04(0.00-2.19)	0.05(0.00-1.86)	0.02±0.10
	D_max_ (Gy)	26.75(0.08-27.47)	26.65(0.06-27.82)	0.16±0.79*	26.42(0.06-27.33)	26.17(0.01-27.13)	0.26±0.74
LV	D_50_ (Gy)	0.34(0.00-16.86)	0.94(0.00-17.56)	-0.71±1.37**	0.24(0.00-6.22)	0.29(0.00-6.98)	-0.09±0.22
	D_max_ (Gy)	26.98(10.86-27.55)	27.26(8.94-28.18)	0.28±1.41*	26.48(1.14-27.31)	26.50(0.05-27.38)	0.20±0.61
LVW	D_50_ (Gy)	0.08(0.00-1.91)	0.11(0.00-1.99)	-0.11±0.22*	0.04(0.00-0.58)	0.04(0.00-0.70)	-0.03±0.07
	D_max_ (Gy)	27.03(18.72-27.41)	27.31(16.36-30.12)	0.42±2.83	26.94(13.07-27.37)	26.67(6.97-27.93)	0.67±1.82
RA	D_50_ (Gy)	0.01(0.00-6.36)	0.02(0.00-8.36)	-0.20±0.57**	0.08(0.00-2.71)	0.01(0.00-2.53)	0.00±0.09
	D_max_ (Gy)	18.22(0.00-27.69)	16.23(0.00-27.96)	0.60±1.45	12.41(0.00-27.34)	7.53(0.00-26.93)	2.03±2.95**
RV	D_50_ (Gy)	0.06(0.00-4.65)	0.15(0.00-7.72)	-0.52±1.06**	0.02(0.00-2.13)	0.02(0.00-2.54)	-0.04±0.12
	D_max_ (Gy)	26.92(6.87-27.48)	27.07(5.57-27.64)	0.45±1.10	26.63(3.88-27.33)	26.45(3.82-27.21)	0.24±0.25**
PA	D_50_ (Gy)	3.65(0.00-17.18)	2.21(0.00-19.14)	-0.17±1.82	1.99(0.00-11.46)	1.47(0.00-7.52)	0.55±1.30
	D_max_ (Gy)	26.95(0.10-27.34)	27.04(0.00-28.33)	0.17±1.24	26.86(0.00-27.04)	26.39(0.00-27.32)	0.21±0.59
Aorta	D_max_ (Gy)	27.04(0.01-27.55)	26.83(0.01-27.74)	0.42±1.2	26.72(0.01-27.34)	26.47(0.00-27.38)	0.31±0.61
HE	D_50_ (Gy)	0.04(0.00-0.43)	0.09(0.00-0.67)	-0.09±0.11**	0.03(0.00-0.19)	0.04(0.00-0.25)	-0.02±0.02**
ESO	D_max_ (Gy)	2.36(0.00-15.31)	2.09(0.00-14.53)	0.04±0.67*	1.48(0.00-11.76)	1.39(0.00-11.85)	0.44±1.49
Lungs	D_5_ (Gy)	5.37(0.34-10.23)	4.78(0.38-8.99)	0.81±2.57	3.17(0.33-7.29)	2.84(0.31-6.92)	0.29±0.20*
	V_7_ (cc)	124.6(32.8-241.2)	112.5(27.5-220.6)	19.45±5.61**	89.3(23.1-187.4)	78.1(20.9-178.3)	7.47±3.24**

4DCT, Four-dimensional computed tomography; 4DcCT, Four-dimensional cardiac computed tomography; ESO, Esophagus; HE, heart excluding CTV; LA, Left atrium; LV, Left ventricle; LVW, Left ventricular wall; PA, Pulmonary arteries; RA, Right atrium; RV, Right ventricle.

For OARs, the metrics are expressed as means with the ranges in parentheses. Discrepancy: Difference between the 3D and 4D dynamic doses (discrepancy=3D dose−4D dynamic dose). A single asterisk (*) indicates a p-value less than 0.05, signifying statistical significance, whereas a double asterisk (**) denotes a p-value less than 0.01, indicating a higher level of statistical significance. The dose to the spinal cord was <0.01 Gy and thus excluded from the statistical analysis.

In the 4D dynamic dose under respiration, the LV, LVW, and RV exhibited maximum doses significantly exceeding the D_max_ of the corresponding IRV (*p*< 0.05), whereas only the LV slightly increased in 4DcCT (*p*> 0.05). IRV tended to significantly overestimate the maximum dose to OARs and metrics such as V_7_ and D_5_ of the lungs (*p<* 0.05), posing challenges for treatment planning in some cases.

#### 3.2.2 Evaluation of dose uncertainties induced by interplay effects in the OARs

**Figure 4:**
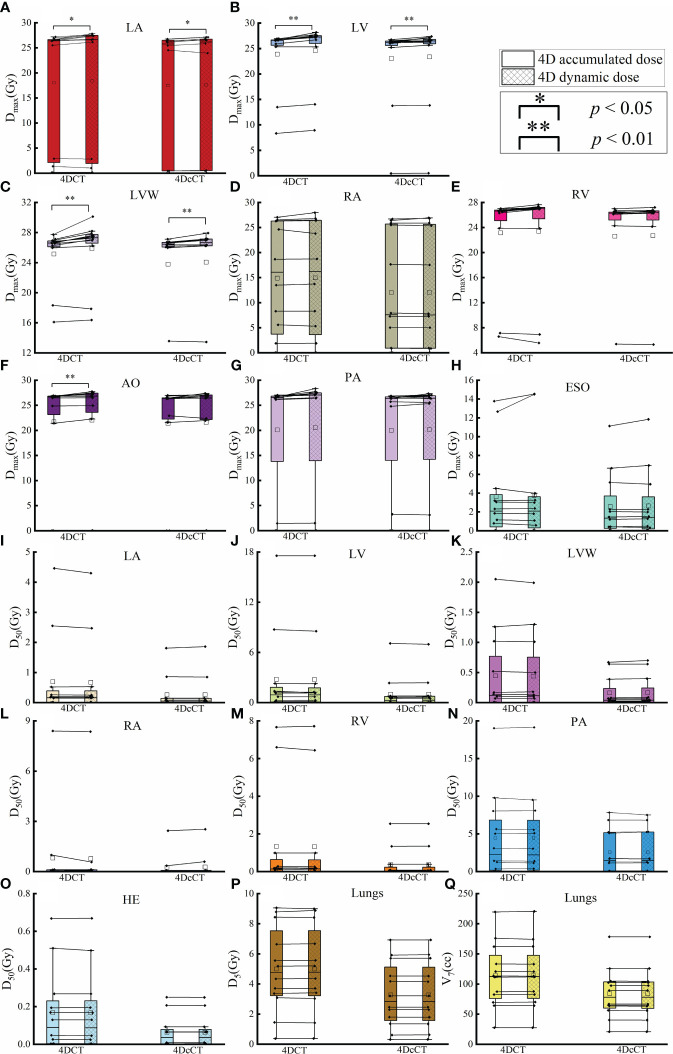
Comparison of dose metric differences in OARs between 4D accumulated and dynamic doses. The figure details the maximum dose (D_max_) and other specific dose metrics for various OARs, including **(A)** D_max_ of LA, **(B)** D_max_ of LV, **(C)** D_max_ of LVW, **(D)** D_max_ of RA, **(E)** D_max_ of RV, **(F)** D_max_ of AO, **(G)** D_max_ of PA, **(H)** D_max_ of ESO, **(I)** D_50_ of LA, **(J)** D_50_ of LV, **(K)** D_50_ of LVW, **(L)** D_50_ of RA, **(M)** D_50_ of RV, **(N)** D_50_ of PA, **(O)** D_50_ of HE, **(P)** V_7_ of lungs, and **(Q)** D_max_ of LA. A single asterisk (*) indicates a *p*-value less than 0.05, signifying statistical significance, whereas a double asterisk (**) denotes a *p*-value less than 0.01, indicating a higher level of statistical significance.

The interplay effect only significantly impacted the D_max_ of several OARs, corroborating the hot spots previously noted (**Figure 4**). In 4DCT, the D_max_ of LA, LV, LVW, and AO increased by an average of 0.39 Gy (*p<* 0.05), 0.68 Gy (*p<* 0.01), 0.72 Gy (*p<* 0.01), and 0.36 Gy (*p<* 0.01), respectively. The D_max_ of LA, LV, and LVW had a significant mean increase of 0.16 Gy (*p<* 0.05), 0.30 Gy (*p<* 0.01), and 0.28 Gy (*p<* 0.01) in 4DcCT. While the maximum doses for other OARs increased slightly, these changes were not statistically significant, and metrics such as V_7_ and D_5_ for the lungs remained almost unchanged (*p*> 0.05). Generally, respiratory motion causes greater dose uncertainties than cardiac pulsations in the OARs.

### 3.3 Evaluation of the estimation accuracy of IRV and OAR_real_ for 4D dynamic dose in OARs

**Figure 5:**
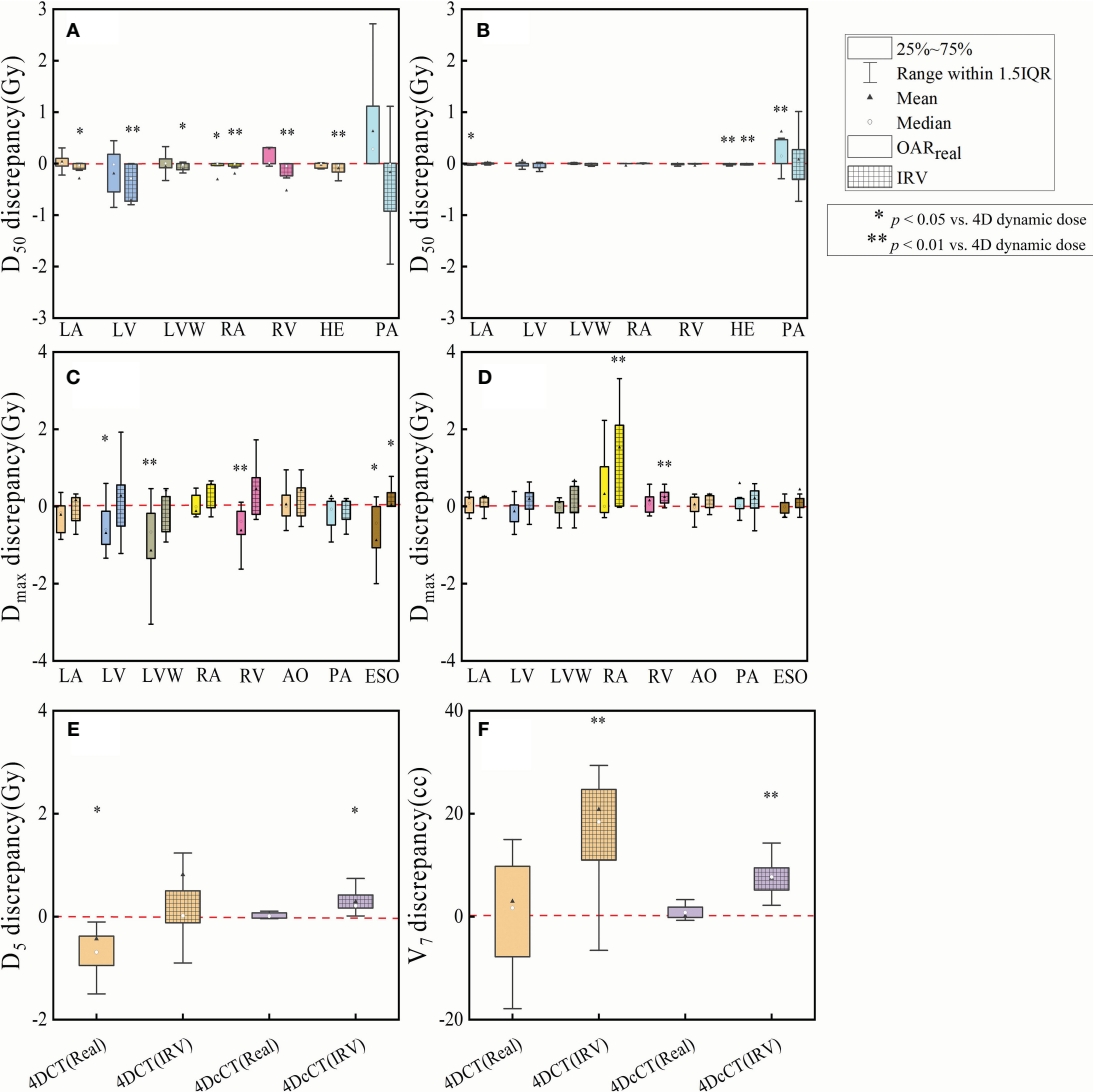
Discrepancies in dose metrics between 3D dose and 4D dynamic dose for OAR_real_ and IRV. The discrepancies are analyzed for **(A)** D_50_ discrepancies in 4DCT, **(B)** D_50_ discrepancies in 4DcCT, **(C)** D_max_ discrepancies in 4DCT, **(D)** D_max_ discrepancies in 4DcCT, **(E)** D_5_ discrepancies in lungs, and **(F)** V_7_ discrepancies in lungs. A single asterisk (*) indicates a *p*-value less than 0.05, signifying statistical significance, whereas a double asterisk (**) denotes a *p*-value less than 0.01, indicating a higher level of statistical significance.

The doses of IRV and OAR_real_ were calculated and compared with the 4D dynamic dose to determine their ability to evaluate the actual dose. The discrepancies between the 3D static and 4D dynamic doses are shown in **Figure 5**. The comparison of dose metrics between the 3D dose and the 4D dynamic dose for IRV and OAR_real_ is detailed in **Supplementary Figure S1** and **Supplementary Figure S2**, respectively.

For IRV, the D_50_ was generally significantly lower than the 4D dynamic dose in 4DCT owing to the volume increment, except for PA. No significant changes were observed in most OAR_real_ apart from the RA. Consequently, significant discrepancies of up to 3 Gy were observed in the PA. However, in 4DcCT, IRV and OAR_real_ demonstrated fewer discrepancies from the 4D dynamic dose, although significant discrepancies were still observed in PA. This may be due to the overlap of the PA with the substrate target in most patients, resulting in a high dose gradient within the PA and greater variability in the D_50_ during cardiorespiratory motion.

Regarding D_max_, the OAR_real_, including the LV, LVW, RV, and ESO, tended to underestimate the actual dose, with significant discrepancies in 4DCT. Conversely, the IRV generally aligned closely with the 4D dynamic dose, except for ESO (*p<* 0.05). In 4DcCT, the dose of OAR_real_ did not significantly deviate from the 4D dynamic dose. However, IRV often overestimated D_max_, with significant overestimations observed in the RA and RV (*p<* 0.01).

Regarding dosimetric indicators of the lungs, IRV introduced an increase in V_7_ (*p<* 0.01), whereas OAR_real_ did not significantly differ from the 4D dynamic dose in 4DCT and 4DcCT. In 4DCT, the D_5_ of OAR_real_ showed a significant variation (*p<* 0.05), whereas IRV did not, possibly because of the substantial difference in the lungs between reference-phase CT and AIP CT. However, in 4DcCT, the dose of OAR_real_ and the 4D dynamic dose in 4DcCT did not significantly differ owing to fewer lung variations during cardiac pulsation. In contrast, IRV tended to overestimate D_5_ because of the increased lung volume overlapping with the heart (*p<* 0.05).

## 4 Discussion

For patients with refractory arrhythmia, the dose uncertainties from respiratory motion were generally greater than those from cardiac pulsation. The interplay effect can deteriorate dosimetric parameters in the substrate target, particularly in the form of cold and hot spots (**Figure 3**). Scattered hot spots for OARs can lead to significant changes in the maximum dose for certain OARs. Uncertainties arising from combining blurring and interplay effects in the cardiorespiratory motion are not effectively mitigated by the ITV-based motion-encompassing technique; large dose discrepancies remain observed in some patients. Additionally, IRV or OAR_real_ did not accurately assess the actual OAR dose.

IMPT-based cardiac radioablation has significant potential compared with CSBRT using photon therapy. The ability to selectively spare these critical structures presents significant advantages in clinical outcomes. It may even pave the way for re-irradiation, which may be necessary considering that catheter ablations are often performed repeatedly (34). Animal (35) and preliminary clinical experiments (36) have been conducted to explore the feasibility of particle therapy in cardiac ablation, with positive results. However, proton therapy is more sensitive to changes in water-equivalent depths penetrated by range uncertainties and anatomical changes (37) and dose uncertainties induced by complicated motion (38). The interplay effects are the interference between the scanning beam spots and intrafractional motion, severely degrading the plan quality (39). The 4D dose reconstruction approach is widely used to evaluate dose uncertainties induced by motion; its feasibility and accuracy have been experimentally validated through various phantom studies (40).

To our knowledge, no studies are available on the dosimetric effects of cardiorespiratory motion in patients undergoing IMPT. However, some studies have mainly focused on virtual ablation targets on 4DCT images of patients with lung cancer (41). Here, we innovatively used 4DCT and 4DcCT images to determine the dosimetric effects of respiratory motion and cardiac pulsation. Furthermore, 4DcCT is highly recommended for a precise CSBRT. This technique involves a retrospective electrocardiographically gated approach combined with contrast-enhanced scanning, clearly visualizing morphological and volumetric changes in the cardiac substructures throughout the patient’s cardiac cycle.

Our study revealed significantly degraded dose metrics for 4D dynamic doses, accurately representing the dose delivered during treatment. The ITV-based motion-encompassing technique could not reduce dose uncertainties owing to combined dose blurring and interplay effects caused by cardiorespiratory motion. Dose uncertainties caused by respiratory motion led to 11 of 12 patients failing to meet the clinical requirements for V_25_ and D_95_. In contrast, cardiac pulsation also caused 6 of 12 patients to fail to meet the clinical requirements. The maximum dose of the target substrate and certain OARs increased in some patients, indicating significant hot spots. These dose uncertainties can also reduce plan quality and result in unnecessary complications. Hence, in the clinical implementation of proton cardiac ablation, additional motion management techniques and strategies to mitigate dose uncertainties stemming from cardiorespiratory motion should be further explored (42).

The interplay effects resulted in significant dose uncertainties in respiratory motion and cardiac pulsation. Generally, these interplay effects increase the maximum dose and decrease the minimum dose, compromising the homogeneity, conformity, and dose-fall-off gradient of the substrate target. This result aligns with previous findings, where interplay effects yielded significant cold and hot spots, influencing the overall dose distribution (43–45). The influence of the interplay effects is greater on respiratory motion, mainly because of the larger motion amplitude during respiration. Widesott et al. (41) similarly demonstrated significant interplay effects, leading to cold spots in the target and hot spots > 110% of the prescribed dose. Regarding the OARs, the interplay effects increased the maximum dose of the LA, LV, and LVW, which is closely associated with primary beating and contraction of the heart occurring in the LV.

In the conventional treatment of moving tumors, OARs are usually contoured directly on AIP CT, whereas our study employed the concept of IRV to evaluate OAR dose (46, 47). The differences between OAR_real_ and IRV were also compared. The results demonstrated that IRV offers a more precise evaluation of the actual dose to each OAR than OAR_real_ in terms of D_max_. However, for some metrics closely related to the volume, such as D_50_, both methods performed poorly. Given the increased IRV due to motion, employing absolute volume metrics such as D_5cc_ for evaluation is advisable. Nevertheless, IRV still fails to predict the actual dose of OARs in many patients, particularly in 4DCT. It often tends to overestimate the maximum dose to OARs, complicating subsequent plan design and optimization. Therefore, a 4D reconstructed dose approach is necessary to evaluate dose uncertainties due to cardiorespiratory motion (24, 48).

This study has limitations. Notably, all research conducted and the results presented are based on simulated data. The temporal structure of the delivery relies on existing research models, which could differ from actual log files. Additionally, our reliance on standard CT scans instead of spectral CT introduced inaccuracies in calculating the Stopping Power Ratio, leading to increased range uncertainties in proton dose delivery (49). Our consideration of motion-pattern uncertainties was limited by technical constraints, with all proton plans initiated at the 0% phase, assuming constant respiratory and cardiac cycles. The starting phase affects the 4D dynamic dose, particularly in hypo-fraction treatments (13, 25, 44), and patients with arrhythmias exhibit respiratory and heart rate variabilities, making fixed-cycle subplans potentially inaccurate. Furthermore, the accuracy of our analysis is also impacted by the DIR algorithm used, which is a critical factor in precision. Future studies will delve deeper into the impact of DIR algorithms. Our analysis also omitted other uncertainties, such as setup, range, and biological dose uncertainties that influence cardiorespiratory motion effects. Future studies will focus on patient-specific motion cycles from real-time position management and ECG data for more precise 4D dose reconstruction and assess the feasibility of robust optimization (50, 51) and rescanning techniques (42, 52) to reduce motion-related dose uncertainties.

## 5 Conclusions

Complex cardiorespiratory motion can introduce dose uncertainties into IMPT implementation in CSBRT. The interplay effects induce hot and cold spots, severely deteriorating the plan quality. The interplay effects are more pronounced during respiration than during cardiac pulsation. Conventional ITV-based motion-encompassing techniques cannot effectively compensate for the dose deviations. IRV and OAR_real_ have inherent challenges in evaluating the actual OAR dose. Therefore, individualized 4D dose evaluation is recommended to ensure the efficacy and safety of CSBRT.

## Data availability statement

The raw data supporting the conclusions of this article will be made available by the authors, without undue reservation.

## Ethics statement

The human study was approved by the Chinese Clinical Trial Registration Center with registration number ChiCTR2200060436. The study was conducted in accordance with local legislation and institutional requirements. Written informed consent was obtained from the individual(s) for the publication of any potentially identifiable images or data included in this article.

## Author contributions

WW: Data curation, Formal analysis, Validation, Writing – original draft. ZL: Conceptualization, Formal analysis, Software, Validation, Writing – original draft. QX: Data curation, Funding acquisition, Methodology, Supervision, Writing – original draft. GW: Conceptualization, Software, Validation, Writing – review & editing. HH: Methodology, Resources, Writing – review & editing. DL: Data curation, Validation, Writing – review & editing. LC: Data curation, Formal analysis, Writing – review & editing. JL: Data curation, Formal analysis, Writing – review & editing. XZ: Data curation, Methodology, Software, Writing – review & editing. TQ: Data curation, Formal analysis, Validation, Writing – review & editing. YS: Methodology, Supervision, Writing – review & editing. GL: Data curation, Methodology, Supervision, Writing – review & editing. SB: Funding acquisition, Investigation, Methodology, Supervision, Writing – review & editing.
