# Supplementary material for: Quantifying dose uncertainties resulting from cardiorespiratory motion in intensity-modulated proton therapy for cardiac stereotactic body radiotherapy
Source: Front Oncol. 2024 Jul 8;14:1399589. doi: 10.3389/fonc.2024.1399589 (PMC11260676; doi:10.3389/fonc.2024.1399589)
Supplement: Supplementary file 1 [file DataSheet_1.docx]

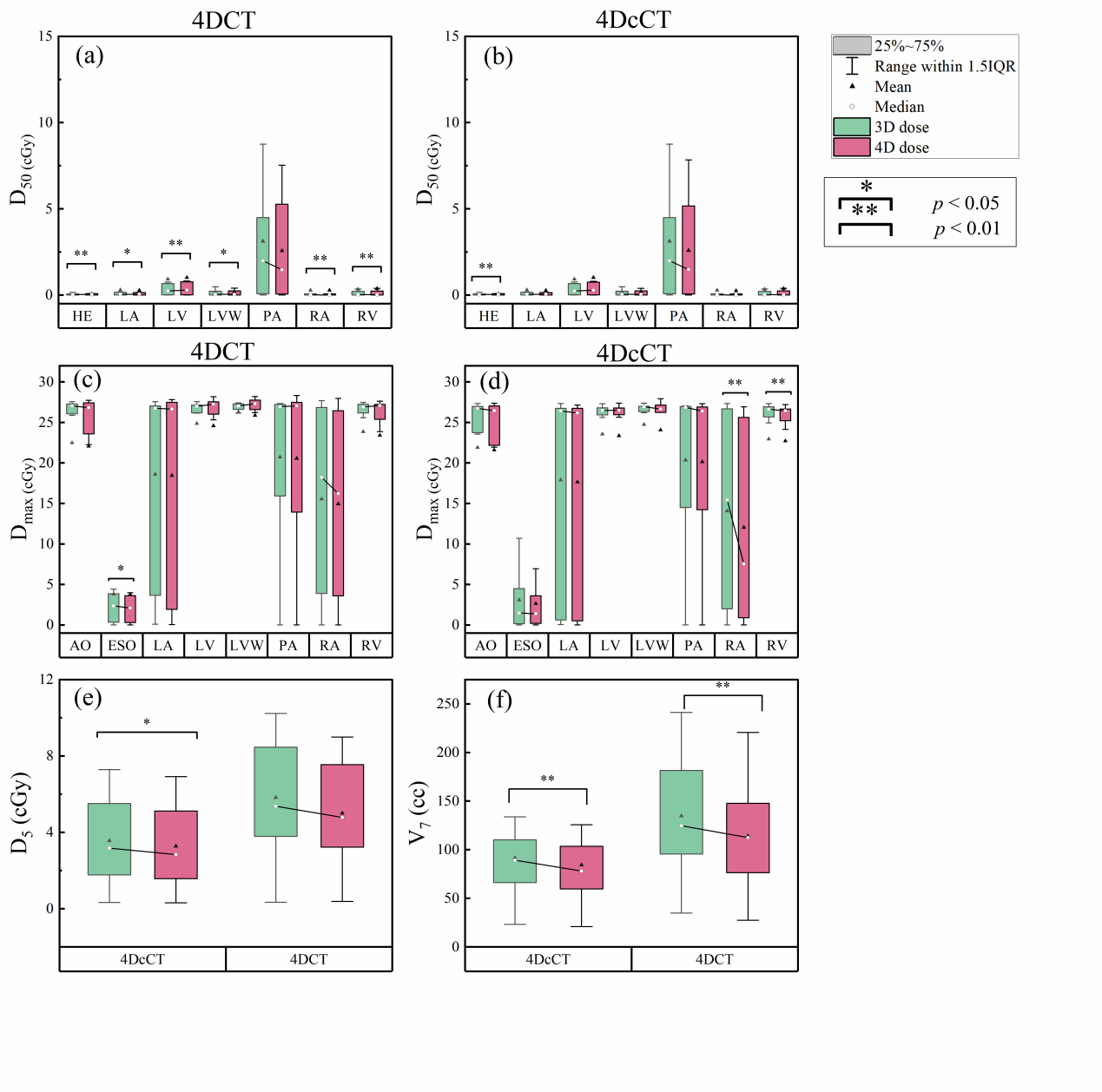


**Figure S1 Comparison of dose metrics between 3D dose and 4D dynamic dose for IRV**

(a) D_50_ comparison in 4DCT, (b) D_50_ comparison in 4DcCT, (c) D_max_ comparison in 4DCT, (d) D_max_ comparison in 4DcCT, (e) D_5_ comparison in lungs, and (f) V_7_ comparison in lungs. A single asterisk (*) indicates a *p*-value less than 0.05, signifying statistical significance, while a double asterisk (**) denotes a *p*-value less than 0.01, indicating a higher level of statistical significance.


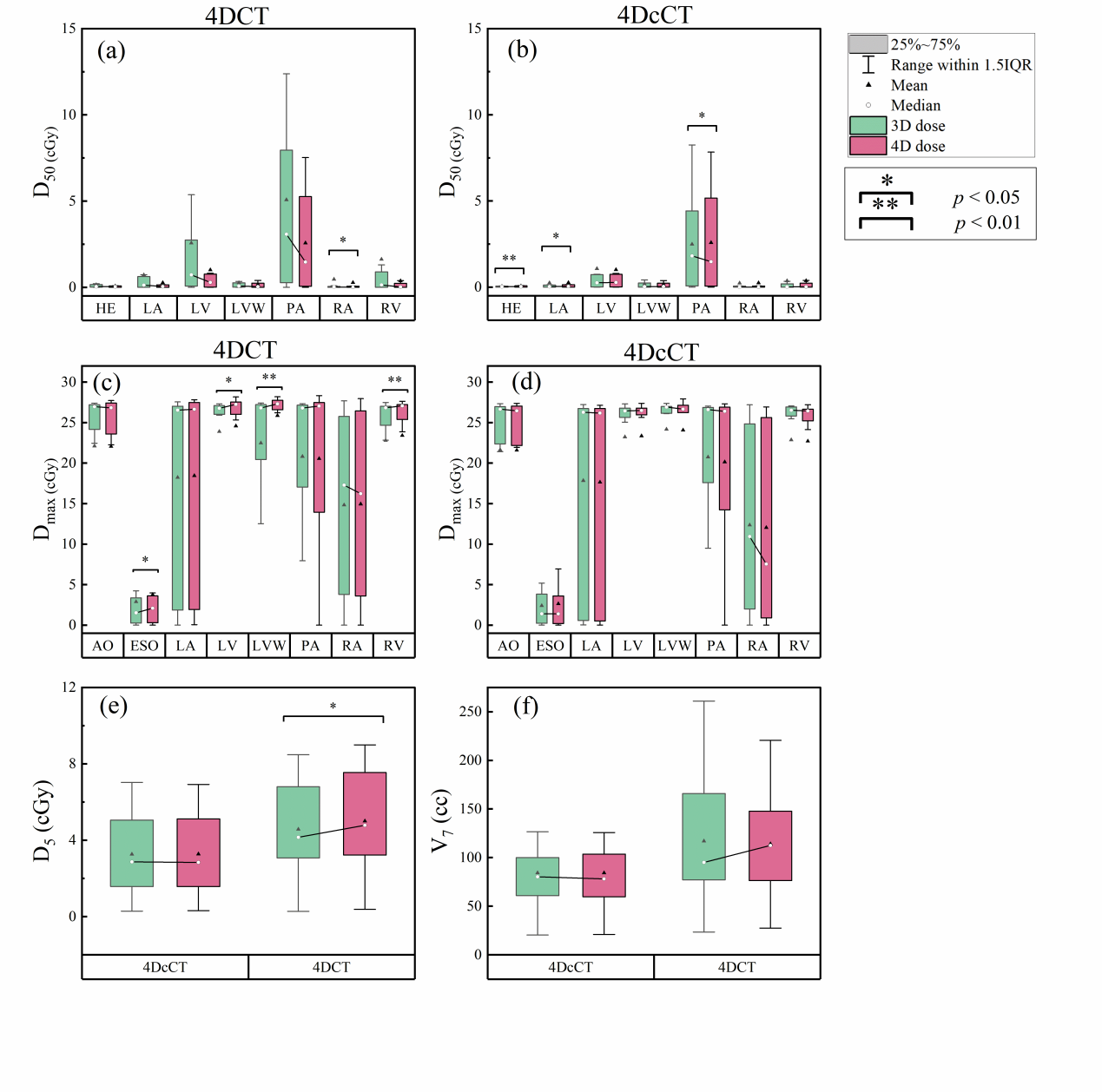


**Figure S2 Comparison of dose metrics between 3D dose and 4D dynamic dose for OAR_real_**

1. D_50_ comparison in 4DCT, (b) D_50_ comparison in 4DcCT, (c) D_max_ comparison in 4DCT, (d) D_max_ comparison in 4DcCT, (e) D_5_ comparison in lungs, and (f) V_7_ comparison in lungs. A single asterisk (*) indicates a *p*-value less than 0.05, signifying statistical significance, while a double asterisk (**) denotes a *p*-value less than 0.01, indicating a higher level of statistical significance.


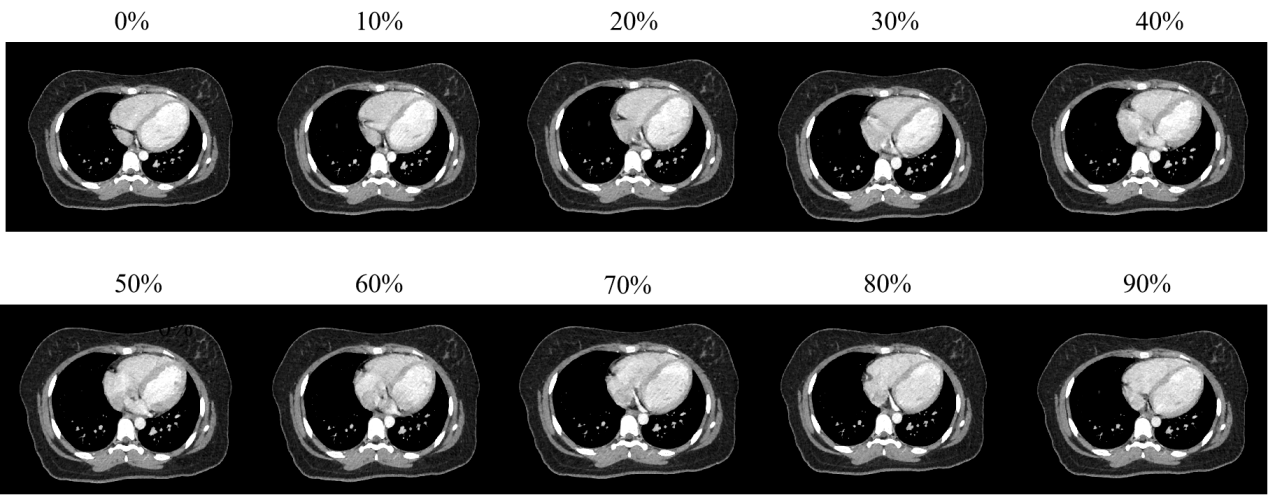


**Figure S3** **The 10 phases of 4DcCT images for one patient**


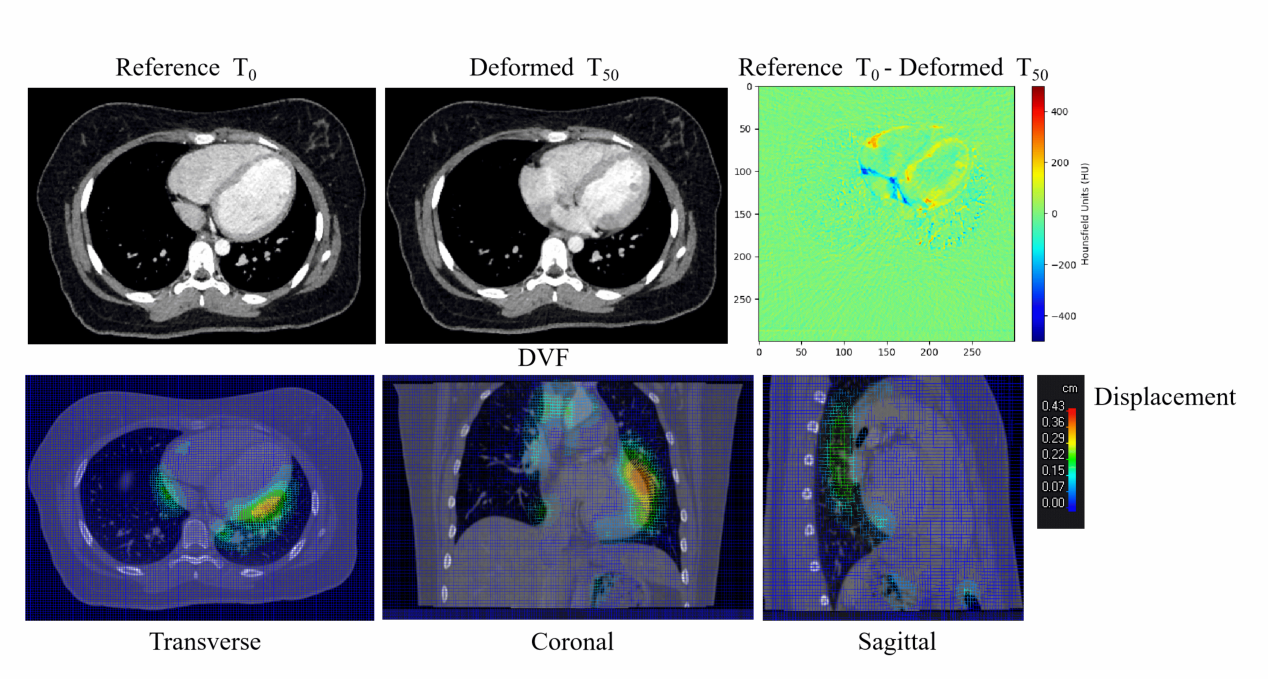


**Figure S4 The results of deformable registration and deformation vector fields between the 0% and 50% phases of one patient's 4DcCT scan**
